# The Dying

**Published:** 1882-10

**Authors:** 


### THE DYING.

"MOTHER, I can see a great distance," said a good man once, as he was just entering on the endless journey. "Ye shall see Heaven open, and the angels of God ascending and descending," was the promise to the Disciples of our Lord, and, through them, to Christians of all time. Literally, as well as metaphorically, in life, as well as in a dying hour, has the declaration being verified—verified every day in the life, as well as in the death, of the righteous; and will be, until death shall be no more. Sights have been seen, and sounds heard—sights and sounds, freighted with ravishing sweetness to Christian people, in the broad daylight of life and health, and in the gloom of the grave; sights and sounds vouchsafed to cheer, when cheer is the most needed, when none can come from any mortal source, as if the very last, last moment of a Christian's life should be a fulfillment of a promise given by the Master, to be with them when they were walking "through the Valley and the Shadow of Death."

The utterances of dying Christians indicate that they see, or think they see, angelic forms, and the familiar faces of the departed dead, hovering about them, and, with smiles of ineffable sweetness, beckon them away to the elysium of the blessed. If these be mere fancies, they are delicious fancies; if facts, they are glorious beyond expression. Whether it were but a dreaming or a seeming, the angels on the ladder from earth to Heaven, and the promise of the Lord, who stood at its top, the preciousness of it was to Jacob all the same, as if it had been an embodied fact, especially as the promise which he heard in his dream was literally complied with.

The film which covers the mortal eye, and hides from physical sense the beings and the things at hand, has been brushed away in the case of patriarchs and prophets of olden time; and later, on the Mount of Transfiguration; and various martyrs of after ages have had their faces so lighted up with heavenliness, that it is difficult to be accounted for, except by the fact of an actual sight of heavenly things.

But, further on in the act and article of dissolution, the sight that pierces ether, faints and fails and fades, and taste is dead, and touch is dead, and tongue, and feeling, and smell, all, all are dead. Not so the ear; it survives them all, for it is the last sense that dies; and it is the repeated testimony of those who have returned to life from the furthest limits beyond, that the whole atmosphere seemed to be filled with sounds so ravishing, as to be indescribable by mortal words. It has been testified to by persons who have been drowned, and then brought to, that the very last perception was that of delightful music.

A dying man sheds no tears. He calls his wife and children, his parents, his best friends, to his bed-side, and, though tear-drops rain from every eye, the contamination of tears never comes to him, never the one falls down his cheek. This is because the manufactories of life have stopped forever ; the human machine has run down at last; every gland of the system has ceased its functions, and that is why death steps in, and, like a remorseless sheriff, takes possession and stops everything. In almost all diseases, the liver is the first manufactory that stops work, one by one the others follow, and all the fountains of life are, at length, dried up; there is no secretion anywhere; the lips and tongue, how dry, as we have all seen; the skin, how dry; or, if moistened by the damp of death, it is from mechanical causes. So the eye in death weeps not; not that all affection is dead in the heart, but because there is not a tear-drop in it, any more than there is moisture on the lip, which undying affection, when it can do nothing else, laves incessantly with the little mop, or feather.

There is one sign of approaching dissolution. We have never seen it alluded to, and yet we have never seen it fail. When the extremities are cold, and the head, the very last part to lose all power of motion, is turned incessantly and quickly and restlessly from one side on the pillow to the other, death comes within an hour. It is worth the effort of a life-time, to be able to die well, to die at a good old age, in peace with all mankind, and in a well grounded faith of an immortal life beyond.

